# Effects of Simulated Airplane Cabin Noise on In-Flight Meal Perception in the Brain Using Electroencephalography

**DOI:** 10.3390/foods13071012

**Published:** 2024-03-26

**Authors:** Manyoel Lim, Sang Sook Kim, Cho-Long Lee, Youngseung Lee, Han Sub Kwak

**Affiliations:** 1 Food Processing Research Group, Korea Food Research Institute, Wanju-gun 55465, Republic of Korea; manyoell@kfri.re.kr (M.L.); sskim@kfri.re.kr (S.S.K.); l.cholong@kfri.re.kr (C.-L.L.); 2 Department of Food Science and Nutrition, Dankook University, Cheonan-si 31116, Republic of Korea; youngslee@dankook.ac.kr; 3 KFRI School, University of Science and Technology, Wanju-gun 55465, Republic of Korea

**Keywords:** food perception, background noise, in-flight meal, electroencephalography, event-related potential

## Abstract

Auditory distractions can impair the sensory evaluation of food; however, the specific impact of airplane cabin noise on the sensory perception of in-flight meals remains poorly studied. Here, we investigated the effects of airplane cabin noise on the visual processing of in-flight meal stimuli using electroencephalography (EEG) in twenty healthy male subjects. Resting-state EEG and event-related potential (ERP) responses to in-flight meal images were acquired during quiet and simulated cabin noise conditions. Participants reported mild discomfort and some loss of appetite when exposed to airplane cabin noise. The analysis of resting-state EEG showed an increase in the absolute power of theta and beta frequency bands in the left superior parietal and left frontal/right central regions under simulated cabin noise conditions, compared to quiet conditions. The ERP results showed that the amplitude of responses evoked by visual meal images in the superior parietal area was reduced in the noise condition compared to the quiet condition. Our findings suggest that airplane cabin noise disrupts the visual perception and attentional processing of in-flight food stimuli. These neural changes imply an impact on integrating sensory information, resulting in altered sensory evaluations of food during in-flight dining experiences.

## 1. Introduction

Food perception is affected by various intrinsic and extrinsic factors [1,2]. When consumers perceive food products, what they smell and taste are intrinsic factors. Extrinsic factors that affect food perceptions include the color of the food, the atmosphere, temperature, pressure, etc. Background sounds or noise have also been found to influence the perception of food products [3]. To date, various food perception studies involving background noise have been conducted using sensory evaluation techniques, in which responses from the five human senses are used to evaluate food and other consumer products [4,5,6,7]. It was reported that the intensity ratings of taste-related attributes such as sweetness and saltiness decreased significantly under a loud noise condition (75–85 dB); however, intensity ratings of crunchiness, a sound-related attribute, were perceived to be higher in the loud noise condition [5]. Under extreme noise conditions, human taste sensations perceived lower intensities of sweetness and higher intensities of umami than in a quiet condition [8]. In music and storytelling conditions, the ability to evaluate alcohol intensity was reduced [4]. Noise conditions also decreased the ability to differentiate among odors [9]. Velasco et al. (2014) showed that white noise conditions reduced liking for and perceived sweetness of six different odor samples [10]. Of the types of background noise, classical music increased the liking of sweet food items, but decreased the liking of savory food items [11].

Among various situations for measuring human responses to sound and noise, the airplane cabin is a unique environment for investigating food perception. Passengers are in a confined, noisy, dry, and low-pressure cabin. These conditions significantly affect appetite and taste perception for foods [12,13,14]. Research has shown that under low atmospheric pressure conditions, individuals exhibit higher thresholds for salt, sugar, glutamate, and most odorants, with the exception of organic acids and bitter tastants [15]. Building on this understanding, Holthuysen et al. conducted a consumer acceptance test of airplane meals in a laboratory, a simulated airplane, and actual airplane conditions [16]. Their findings revealed no difference in the overall liking of two meal types in the laboratory setting, whereas a significant difference was observed in both the simulated and actual airplane settings. This highlighted the impact of consumption contexts on meal acceptance but did not specifically address the effects of background airplane noise on taste perception [16].

Visual food perception that occurs before the evaluation of the odor and taste of food products is the primary pathway for information processing about given foods [17]. After visual evaluation, humans recognize foods by smelling and tasting them. However, most sensory scientists have not conducted studies on visual food perception because it occurs prior to food tasting and is difficult to measure using traditional sensory evaluation methods. Currently, various new technologies are being applied for sensory evaluation, such as 3D virtual reality, eye-trackers, and face-readers [18]. Among them, electroencephalography (EEG) can be used to measure brain activity associated with visual food perception with high temporal resolution [19]. Previous visual event-related potential (ERP) studies have revealed the spatiotemporal brain dynamics that mediate the evaluation of the energetic content of foods, judgments about meal portion sizes, and liking of diverse meal compositions [20,21,22,23]. Accumulating evidence indicates that visual food stimuli activate multiple brain regions involved in visual and attentional processing, sensory input integration, subjective reward value encoding, and decision-making processes [17,21,24]. Thus, by studying these neural responses to food images, we can better understand how auditory distractions, such as cabin noise, might alter the perceived appeal of food, independent of the direct gustatory experience.

Understanding sensory integration and neural mechanisms of perception in the context of in-flight meal experiences is important, as these are not solely about taste but encompass a multisensory experience where sound, sight, and smell play significant roles [7,25]. Investigating how airplane cabin noise, a common environmental factor, affects meal perception can inform our understanding of how our senses integrate in complex environments. Furthermore, air travel is a common mode of transportation, and meal service is a significant part of the passenger experience. Identifying how cabin noise affects meal perception could lead to strategies to improve satisfaction, such as modifying the meal presentation or selecting flavors that are less influenced by noise, thereby enhancing passenger satisfaction.

Our study aimed to investigate the neural responses to in-flight meal images under the simulated conditions of loud airplane cabin noise using EEG. By examining both resting-state oscillatory activity and visual responses to meal images, we aimed to elucidate how such environmental noise influences brain activity related to visual attentional processing and how it may affect appetite and meal perception. We hypothesized that exposure to loud airplane cabin noise would result in discomfort among participants and decreased appetite for in-flight meal images. Also, we expected that, during the loud airplane cabin noise condition, participants may exhibit altered spontaneous oscillatory brain activity and a reduced response in regions associated with visual attentional processing.

## 2. Materials and Methods

### 2.1. Subjects

Twenty-two healthy male volunteers (23–40 years of age, mean = 29.5) participated in this study. None of the participants reported neurological or psychiatric disorders. All subjects were right-handed. The study protocol was approved by the Institutional Review Board of Dankook University (DKU 2019-04-021-001). All participants provided written informed consent.

### 2.2. Stimuli

The airplane cabin noise of a Boeing 777 aircraft on YouTube (https://www.youtube.com/watch?v=co7KgV2edvI, accessed on 19 September 2022) was played using home-theater speakers (BZ-T3710, Britz International Co., Ltd., Paju-si, Korea) in a soundproof EEG testing booth (170 cm × 170 cm × 215 cm, width × depth × height, respectively). Two speakers were located 10 cm away from the left and right corners in front of but facing opposite to the subject to generate indirect airplane cabin noise. The intensity of the airplane cabin noise was measured using a sound level meter (Testo 815, Testo SE & Co., Lenzkirch, Germany). The background noise in the booth was approximately 94–98 dB at 10 cm away from the speaker and approximately 80–83 dB around participants’ ears. This intensity of airplane background noise was similar to that in previous studies, which showed 80–85 dB in airplane cabins [7,26]. The quiet condition in the booth was below 35 dB of background noise, which was below the detection limit of the sound level meter (Testo 815, Testo SE & Co.).

### 2.3. Experimental Task

**Figure 1:**
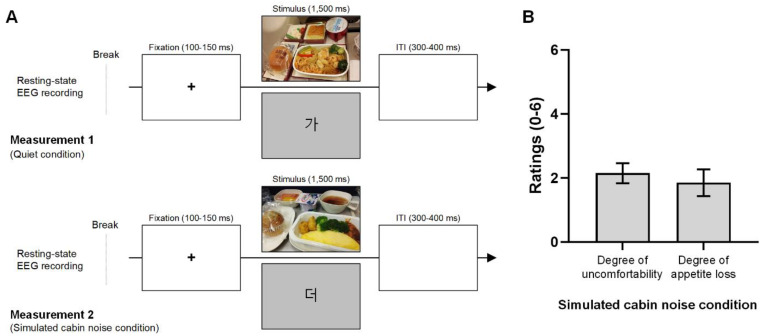
Experimental design of the EEG recording and participant ratings for the noise condition. (**A**). The five-minute EEG recording during the resting state and the visual event-related potential experiment were conducted. Measurement 1, representing the quiet condition, was conducted without simulated airplane cabin noise. Measurement 2 followed the same procedure as Measurement 1 but under airplane background noise (approximately 80 dB). The order of measurements 1 and 2 was counter-balanced. During visual stimulation, participants were shown either in-flight meal photos or Korean characters on the monitor, with a total of 100 stimuli comprising ten meal photos (displayed twice each) and twenty Korean characters (displayed four times each). Each trial started with a 100–150 ms fixation cross, followed by 1500 ms of visual stimuli, and a 300–400 ms intertrial interval. (**B**). Participants rated their level of discomfort (0: no discomfort, 6: extremely uncomfortable) and the extent of appetite loss (0: no appetite loss, 6: extreme appetite loss) in response to the in-flight meal photo under the noise condition. Participants reported slight discomfort with a mean rating of 2.2 and some appetite loss at 1.9 under the noise condition.

The experiment consisted of two measurements (Figure 1A). Measurement 1, representing the quiet condition, was conducted without airplane cabin noise, while Measurement 2 was conducted with simulated airplane cabin noise. Each measurement lasted approximately 12 min and included a three-minute break. The order of measurements 1 and 2 was counterbalanced. Participants were instructed not to eat or drink any food or beverages for two hours before the test.

The first part of each measurement involved a five-minute resting-state EEG recording of participants’ brain activity. This was followed by a visual ERP experiment. The visual stimulation sequence was executed using the E-Prime^®^ extension for Magstim EGI software (version 2.0, Psychology Software Tools, Sharpsburg, PA, USA). During the visual stimulation, participants were presented with either economy class in-flight meal photos or Korean character figures (refer to Supplementary Figures S1 and S2) on the monitor. Korean character figures were used to minimize adaptation to the meal photos. In total, 100 stimuli were presented, with ten in-flight meal photos (20% of the stimuli, each shown twice) and twenty Korean characters (80% of the stimuli, each shown four times) randomly displayed to participants. The size of the in-flight meal photos was 800 × 500 pixels. The Korean characters were displayed on a light gray background. Each trial began with a fixation cross at the center of the screen for 100–150 ms, followed by the display of an in-flight meal photo for 1500 ms. The intertrial interval was 300–400 ms.

After completing Measurement 2 (airplane cabin noise condition), participants were asked to fill out questionnaires assessing the effects of airplane cabin noise experienced during the experiment. These questionnaires included items where participants rated the noise intensity compared to their previous experiences with actual airplane cabin noise (-3: extremely low, 0: same, 3: extremely high). Additionally, they assessed the level of discomfort due to the noise condition (0: no discomfort, 6: extremely uncomfortable), and the degree of appetite loss in response to viewing the in-flight meal photo under the noise condition (0: no appetite loss, 6: extreme appetite loss).

### 2.4. EEG Recordings

The EEGs of each participant were recorded using a 256-channel dense array EEG (Geodesic EEG System 400, MagstimEGI, Eugene, OR, USA) with a high-density Geodesic Sensor Net 200. The net consisted of 256 Ag/AgCl sponge sensors with three different sizes based on head size. The impedance of the electrodes was maintained at less than 50 kΩ prior to the experiment, as recommended by the manufacturer. The EEGs were recorded with a band-pass of 0.1–100 Hz and were continuously collected at 1000 Hz using NetStation 400 version 5.4.2 software (MagstimEGI, Eugene, OR, USA). EEG results from two participants could not be used because of severe head movement during the resting-state EEG. The results from twenty participants were used in further data analyses (23–40 years of age, mean = 29.7).

### 2.5. Resting-State EEG Data Analysis

In terms of the five-minute cerebral activity, the recorded EEG signals for each participant were detrended and digitally filtered using 0.3 Hz high-pass and 100 Hz low-pass filters and a 60 Hz notch filter. The EEG signals for the channels of 10–20 system positions (FP1, FP2, F7, F3, Fz, F4, F8, T3, C3, Cz, C4, T4, T5, P3, Pz, P4, T6, O1, O2, A1, and A2) were extracted from the 256-channel results. The extracted results were processed using the quantitative EEG (qEEG) analysis software, NeuroGuide 3.0.9 (Applied Neuroscience, Inc., Largo, FL, USA). Artifact removal was conducted using automatic editing in the NeuroGuide software. Briefly, the “high” template artifact rejection option was selected. Drowsiness, eye movements, and muscle movements were selected for Z-score artifact rejection with a high-sensitivity option. Approximately 10–12 s of artifact-free, eyes-open EEG segments were manually selected, followed by the application of the automatic selection option to extract at least 60 s of EEG signals from the five-minute recording results. Data from two participants were excluded from further analysis due to not meeting this minimum requirement. Power spectral analysis of the EEG signals was performed using a Fast Fourier transform in various frequency bands: theta (4–8 Hz), alpha (8–12 Hz), and beta (12–25 Hz). Absolute power in both quiet and noisy conditions was calculated to assess the impact of airplane background noise on baseline neural activity, using NeuroGuide 3.0.9 software (Neurostat, Applied Neuroscience, Inc.). Paired two-tailed *t*-tests were then used to compare these power levels between the quiet and noisy conditions. Statistical significance was set at *p* < 0.017 (0.05/3; for three frequency bands), adjusted with the Bonferroni correction.

### 2.6. Event-Related Potential Data Analysis

The EEG data were preprocessed offline using MATLAB 2020 (MathWorks, Natick, MA, USA) and EEGLAB 2021.0 software [27]. First, the data were detrended and digitally filtered using a 0.1 Hz high-pass and a 30 Hz low-pass filter. Bad channels with signals above ±75 µV were deleted and interpolated. The data were then segmented into epochs starting 100 ms prior to stimulus presentation and ending 900 ms post-stimulus. Independent component analysis was performed to remove artifacts. Components with more than 80% chance of an electromyogram, 80% chance of eye movement and blinking, or 40% chance of an electrocardiogram were automatically rejected. The EEG signals were then re-referenced to the mastoid channel. The responses to the in-flight meal stimuli for each subject under the different measurement conditions were averaged. In this study, amplitudes for superior parietal areas (Pz, P3, and P4) in quiet and noisy conditions were compared, as these areas are known to be related to visual and attentional processing in food cues [24]. The peak amplitudes of the EEG response to the in-flight meal image stimuli were identified within 360–420 ms. A paired two-tailed *t*-test was conducted to assess the differences in the mean amplitude of the response to the in-flight meal image stimuli within the 360–420 ms range between quiet and noisy conditions. The threshold for significance was set at *p* < 0.05.

## 3. Results

### 3.1. Participant Survey for Background Noise

All participants included in the final dataset (n = 20) had previously traveled on commercial airplanes and had experience with in-flight meals during flights. The mean ratings for the similarity of airplane cabin noise in this study were −0.2 ± 1.2 (−3: extremely low, 0: same, 3: extremely high). Therefore, the background noise in the experiment was similar to actual airplane cabin noise. Figure 1B shows the degree of discomfort and the degree of appetite loss under the noise condition. The degree of discomfort from the noise was 2.2 ± 1.4 (0: no discomfort, 6: extremely uncomfortable), which suggests the participants were slightly discomforted. The mean rating of the loss of appetite was 1.9 ± 1.9 (0: no appetite loss, 6: extreme appetite loss); therefore, the participants responded some appetite loss under the noise condition.

### 3.2. Resting-State EEG Power

**Figure 2:**
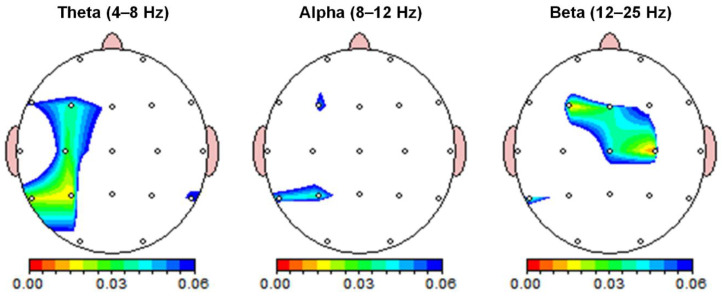
Resting-state spectral power differences. Absolute power between quiet and noise conditions was compared across theta (4–8 Hz), alpha (8–12 Hz), and beta (12–25 Hz) frequency bands. Color-coded maps indicate paired *t*-test *p*-values. Absolute power was significantly higher in the theta and beta bands during noise compared to quiet conditions (*p* < 0.017), with the Bonferroni correction applied for multiple frequency bands.

The absolute power of each sensor for the qEEG analysis is shown in Table 1. Significant differences in absolute power between the quiet and airplane cabin noise conditions were found in the theta and beta frequency bands (Table 1 and Figure 2). Differences between the quiet and noise conditions were observed in the left superior parietal area (P3) for the theta band. The absolute power of the theta band was significantly stronger in the P3 channels under the noise condition (*p* < 0.017). There was no significant difference in absolute power between the quiet and noise conditions in the alpha frequency band (*p* > 0.017). The significant differences in absolute power between quiet and noise conditions were observed in the left frontal (F3) and right central (C4) regions for the beta band. In the beta band, the absolute power was significantly higher in the F3 and C4 channel under the noise condition (*p* < 0.017).

### 3.3. Event-Related Potential Response

**Figure 3:**
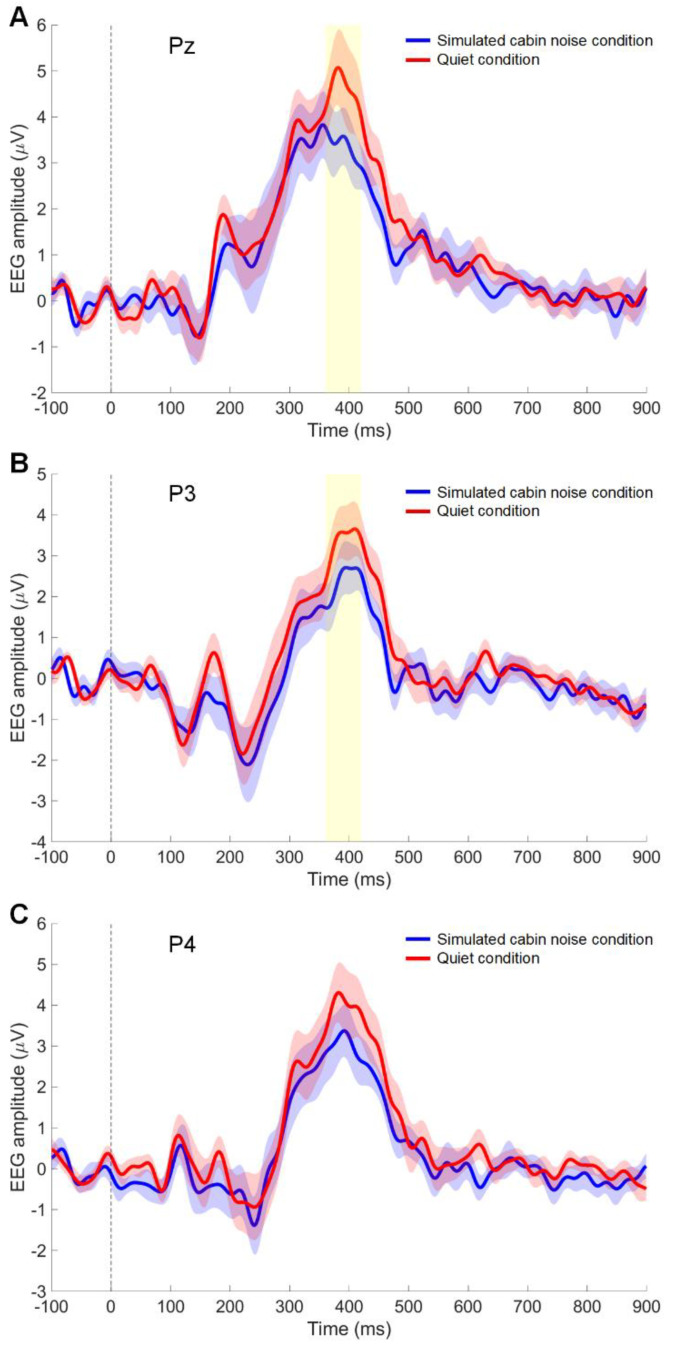
Event-related potentials (ERP) to in-flight meal images. The time course of the grand-averaged ERP reveals the differences in brain responses between quiet conditions (red line) and simulated cabin noise conditions (blue line) at the Pz (**A**), P3 (**B**), and P4 (**C**) channels. The shaded area around the lines represents the standard error of the mean. The yellow highlight from 360–420 ms indicates the time window where significant differences in ERP were observed (*p* < 0.05). The vertical dotted line indicates stimulus onset.

Figure 3 presents the grand-averaged waveform of visual meal image evoked responses from the superior parietal regions. A significant effect of stimulus condition was observed on the amplitude in the superior parietal area (Pz channel) (*p* = 0.040). The average amplitude (mean ± standard deviation) was higher in the quiet condition (4.6 ± 3.5 µV) compared to the noise condition (3.4 ± 2.8 µV) in the Pz channel (Figure 3A). Figure 3B displays the results for the left superior parietal area (P3 channel), where the stimulus condition significantly affected the amplitude in the P3 channel (*p* = 0.007). In the P3 channel, the average amplitude for the in-flight meal stimuli was greater in the quiet condition (3.4 ± 2.8 µV) than in the noise condition (2.1 ± 2.2 µV). Figure 3C shows the grand-averaged waveform in the right superior parietal area (P4 channel). In the P4 channel for the in-flight meal stimuli, the average amplitude in the quiet condition was slightly higher (4.0 ± 3.3 µV) compared to the noise condition (3.1 ± 2.9 µV), but this difference was not statistically significant (*p* = 0.140).

## 4. Discussion

This study examines the effect of loud airplane cabin noise on neural responses to in-flight meal images using EEG. Participants reported mild discomfort and some loss of appetite when exposed to this noise. The findings suggest that the airplane cabin noise used in the EEG booth may have a significant impact on comfort and appetite. The qEEG analysis indicated an increase in the absolute power of the theta and beta frequency bands under airplane cabin noise conditions compared to quiet conditions in the left superior parietal and left frontal/right central regions. The ERP response showed that the amplitude of responses evoked by visual meal images in the superior parietal area was reduced in the noise condition compared to the quiet condition.

The qEEG results revealed higher spontaneous oscillatory brain activity under noise conditions compared to quiet conditions. Since the subjects were not engaged in any specific activities during this period, the observed changes in brain activity might be attributed to the influence of the airplane cabin noise, which was approximately 80 dB. In the noise condition, the absolute power in the theta band in the left superior parietal area was significantly higher than in the quiet condition. Theta waves are often associated with states of drowsiness or the early stages of sleep but can also indicate cognitive processing related to attention and memory encoding [28]. Under noise conditions, which are often perceived as stressful or distracting, the brain might increase theta activity as part of an adaptive response to focus attention or manage the stressor [29]. On the other hand, beta waves are linked to active, alert consciousness and cognitive engagement [30,31]. The absolute power of the beta waves was significantly higher in the superior parietal region under the noise condition than in the quiet condition. This implies that the brain might require more effort or activation to process these images in a noisy environment. While these findings relate to visual processing, they might also suggest an impact on sensory processing, including the alteration of food perception in noisy conditions.

Heightened alertness and cognitive effort, as indicated by the increased theta and beta activity, may redirect cognitive resources towards coping with the immediate environment. This reallocation could detract from the ability to fully engage in the visual presentation of meals. Such a shift might lead to a generalized reduction in appetite, as reported by our participants, where the physiological state induced by noise condition overrides the anticipated pleasure from viewing the meals. Moreover, the increase in oscillatory activity within the superior parietal and frontal regions associated with sensory integration and decision-making indicates that noise-induced stress could impair the cognitive aspects of food evaluation, including the integration of multisensory food cues. This might cause the meals to be perceived as less satisfying under noisy conditions.

The significant differences observed in the Pz and P3 channels between quiet and noise conditions indicate a reduced response in the superior parietal region to in-flight meal images during exposure to simulated cabin noise. The superior parietal lobe plays a critical role in directing attention to relevant stimuli and integrating sensory information to construct a coherent perceptual experience [24,32,33]. The diminished response might reflect a general reduction in visual perception and attentional processing, thereby impairing the integration of sensory inputs from visual meal stimuli under noise conditions. Previous studies have reported reductions in perceptions of sweetness and saltiness [5], intensity of alcohol taste during consumption [4], and odor discrimination [9,10] under noisy conditions compared to quieter environments. These findings suggest a possible link between noise-induced lower brain activity and reduced sensory perception. Consequently, the reduction in superior parietal responsiveness induced by noise could extend to actual sensory experiences, possibly leading to decreased evaluations of taste and odor in real foods.

The integration of visual, olfactory, and gustatory inputs is essential for the full enjoyment of meals [1,34]. Thus, the observed reduction in superior parietal activity could lead to a less immersive dining experience. In practical terms, this might manifest as passengers finding meals less appealing or flavorful, which could dissuade them from choosing certain airlines based on the perceived quality of the in-flight dining experience. Airlines might therefore consider adopting measures such as improving cabin ambiance, optimizing meal presentation, and even adjusting recipes to compensate for alterations in sensory perception induced by noise. Future research could explore these aspects, providing airlines with targeted strategies to enhance the dining experience under varying auditory conditions.

Our study highlights how loud airplane cabin noise affects neural responses to in-flight meal images, but it is also important to recognize the role of real travel conditions, such as time zone changes and stress. These factors can disrupt circadian rhythms and induce jet lag, significantly influencing appetite and meal perceptions. Stress from travel, such as flying anxiety or adjusting to new environments, may further impact eating preferences. The interaction between these conditions and cabin noise could amplify the effects on in-flight meal perceptions. For example, jet lag stress might worsen discomfort and appetite loss, while stress responses could alter sensory perceptions, affecting meal experiences. Understanding these dynamics is crucial for airlines and catering services aiming to improve passenger satisfaction. Future research should investigate how cabin noise, jet lag, and stress together influence meal perception.

This study had several limitations. First, we recruited only male participants, which limits generalizability. This decision was influenced by the complexities associated with the menstrual cycle in women, which can affect neural responses to food stimuli due to hormonal fluctuations [35]. Existing research indicates sex differences in neural responses to food stimuli [36,37], with men and women displaying distinct brain activities in response to visual, auditory, and gustatory food stimuli. Specifically, differences in brain regions related to emotion regulation and eating promotion suggest that these gender-specific variations, including menstrual cycle effects in women, could significantly impact how individuals perceive and evaluate food under various conditions, including loud background noise. By initially focusing on male participants, we aimed to minimize these hormonal variability factors. Future studies should include female participants or both sexes, considering the potential impacts of the menstrual cycle, to better understand the effects of loud background noise on food image perception. Second, the experiment was conducted using in-flight meal images presented on a monitor. This situation is quite different from the presentation of actual food products typically used in sensory testing. While presenting actual food may provide a more direct experience, their repeated presentation in an EEG booth is not feasible due to space and setup limitations. These constraints might negatively affect the quality and reliability of the collected data. Furthermore, our study did not explore participants’ perception of food types other than in-flight-type meals. This limitation raises questions about whether the observed results are influenced more by the auditory conditions or by the inherent characteristics of in-flight meals themselves. Future research should explore how noise affects the perception of various meal types to clarify the roles of environmental noise versus meal-specific factors.

## 5. Conclusions

Our study provides evidence that exposure to airplane cabin noise significantly affects neural processing, as demonstrated by alterations in qEEG and ERP measures. The increased theta and beta band activity in the brain, indicative of heightened arousal and potential stress response under noisy conditions, coincides with a reduction in superior parietal activity during visual tasks. This suggests a decreased ability to effectively perceive and attend to visual stimuli, such as in-flight meal images, in noisy environments. These neural changes imply an impact on integrating sensory information, resulting in altered sensory evaluations of food during in-flight dining experiences. This study not only advances our understanding of the neural mechanisms underlying visual perception of food in varying auditory environments but also highlights the importance of considering environmental noise in the sensory evaluation of foods.

## Figures and Tables

**Table 1:** FFT absolute power values for quiet and noise conditions and comparison by paired *t*-test.

	Theta (4.0–8.0 Hz)			Alpha (8.0–12.0 Hz)			Beta (12.0–25.0 Hz)		
	Quiet	Noise	*p*-Value	Quiet	Noise	*p*-Value	Quiet	Noise	*p*-Value
	Intrahemispheric: left								
FP1	9.4 ± 4.0	12.1 ± 8.9	0.148	7.0 ± 4.6	7.8 ± 5.4	0.085	9.6 ± 6.5	11.8 ± 12.5	0.187
F3	6.5 ± 2.1	8.0 ± 4.7	0.036	6.3 ± 4.6	6.9 ± 5.3	0.049	7.2 ± 3.4	8.2 ± 4.6	0.012 *
C3	4.1 ± 1.2	5.2 ± 3.6	0.030	5.1 ± 3.9	5.6 ± 4.4	0.120	6.1 ± 3.4	6.6 ± 3.4	0.143
P3	2.4 ± 1.1	3.6 ± 3.5	0.015 *	4.5 ± 4.8	5.5 ± 7.5	0.038	4.3 ± 2.6	4.9 ± 3.0	0.088
O1	1.6 ± 0.8	2.2 ± 2.4	0.072	2.9 ± 2.5	3.6 ± 5.4	0.264	3.8 ± 2.5	4.2 ± 3.3	0.243
F7	6.2 ± 1.9	7.5 ± 4.1	0.057	5.8 ± 4.4	6.2 ± 4.7	0.143	8.4 ± 5.6	8.9 ± 5.6	0.295
T3	3.1 ± 1.1	3.6 ± 2.8	0.155	4.2 ± 4.6	4.1 ± 3.7	0.189	9.4 ± 6.9	9.1 ± 7.2	0.867
T5	1.4 ± 0.6	2.1 ± 2.2	0.019	2.5 ± 2.4	2.9 ± 3.6	0.041	2.5 ± 1.0	3.0 ± 1.8	0.033
	Intrahemispheric: right								
FP2	8.9 ± 2.8	12.3 ± 10.9	0.150	7.2 ± 4.6	7.8 ± 5.5	0.208	10.8 ± 5.2	11.6 ± 6.0	0.497
F4	6.6 ± 2.2	7.9 ± 4.7	0.124	6.4 ± 4.4	6.9 ± 5.1	0.139	7.3 ± 2.9	8.1 ± 4.4	0.072
C4	4.4 ± 1.4	5.2 ± 3.4	0.088	5.8 ± 4.4	6.3 ± 5.4	0.357	6.2 ± 2.5	7.3 ± 3.8	0.006 *
P4	2.6 ± 1.1	3.4 ± 3.1	0.093	4.7 ± 4.4	5.6 ± 7.3	0.318	4.3 ± 2.1	4.8 ± 3.2	0.183
O2	1.6 ± 0.7	2.0 ± 2.1	0.172	2.6 ± 2.0	3.1 ± 3.5	0.307	3.3 ± 2.2	4.0 ± 3.3	0.161
F8	6.1 ± 1.8	7.5 ± 4.8	0.141	5.9 ± 4.2	6.3 ± 4.6	0.132	8.2 ± 3.7	8.8 ± 4.8	0.309
T4	3.3 ± 0.9	3.9 ± 2.5	0.091	3.9 ± 2.5	4.0 ± 2.7	0.643	9.9 ± 7.3	9.8 ± 6.0	0.852
T6	1.7 ± 0.7	2.2 ± 2.1	0.054	3.1 ± 2.7	3.4 ± 4.0	0.495	3.3 ± 1.9	3.5 ± 2.5	0.510
	Intrahemispheric: center								
Fz	8.0 ± 3.1	9.7 ± 5.7	0.067	7.1 ± 5.1	7.7 ± 5.7	0.127	7.2 ± 2.8	8.0 ± 3.9	0.036
Cz	6.8 ± 2.2	8.1 ± 4.7	0.095	7.4 ± 5.2	8.2 ± 6.5	0.289	6.9 ± 2.9	7.6 ± 3.7	0.043
Pz	3.4 ± 3.4	3.7 ± 3.0	0.387	3.4 ± 2.6	4.4 ± 5.8	0.099	3.6 ± 2.0	4.2 ± 3.0	0.100

Statistical significance was set at *p* < 0.017 (0.05/3; for three frequency bands), adjusted with the Bonferroni correction. * *p* < 0.017.

## Data Availability

The original contributions presented in the study are included in the article/Supplementary Materials, further inquiries can be directed to the corresponding author.

## Supplementary Materials

The following supporting information can be downloaded at: https://www.mdpi.com/article/10.3390/foods13071012/s1, Figure S1: Ten in-flight meal images; Figure S2: Twenty Korean character images.
