# Supplementary material for: Effects of Simulated Airplane Cabin Noise on In-Flight Meal Perception in the Brain Using Electroencephalography
Source: Foods. 2024 Mar 26;13(7):1012. doi: 10.3390/foods13071012 (PMC11011798; doi:10.3390/foods13071012)
Supplement: Supplementary file 1 [file foods-13-01012-s001.zip › foods-2933957-supplementary.pdf]

Supplementary Figures

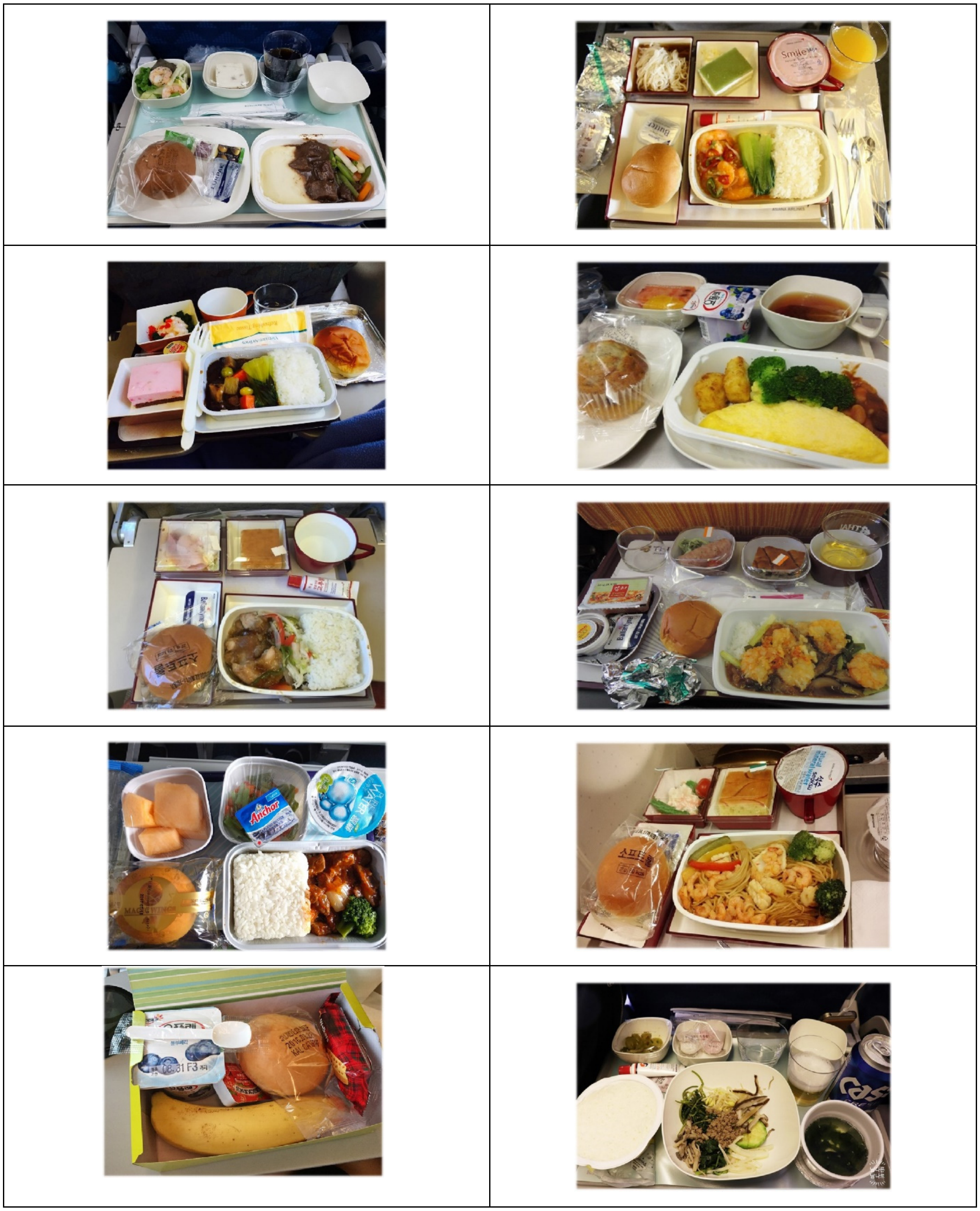

Figure S1. Ten in-flight meal images.

가	누	더	로
므	비	샤	요
자	초	쿠	터
푸	헤	구	디
제	노	토	이

Figure S2. Twenty Korean character images.
